# Scale-up and sustainability of a personalized end-of-life care intervention: a longitudinal mixed-methods study

**DOI:** 10.1186/s12913-021-06241-6

**Published:** 2021-03-10

**Authors:** Alyson Takaoka, Benjamin Tam, Meredith Vanstone, France J. Clarke, Neala Hoad, Marilyn Swinton, Feli Toledo, Anne Boyle, Anne Woods, Erick H. Duan, Diane Heels-Ansdell, Lily Waugh, Mark Soth, Jill Rudkowski, Waleed Alhazzani, Dan Perri, Tania Ligori, Roman Jaeschke, Nicole Zytaruk, Deborah J. Cook

**Affiliations:** 1 Department of Health Research Methods, Evidence and Impact, McMaster University, Hamilton, Ontario Canada; 2 Department of Medicine, McMaster University, Hamilton, Ontario Canada; 3 Department of Family Medicine, McMaster University, Hamilton, Ontario Canada; 4 Department of Critical Care Medicine, St Joseph’s Healthcare Hamilton, 50 Charlton Avenue East, Hamilton, Ontario L8N 4A6 Canada; 5 Department of Spiritual Care, St Joseph’s Healthcare, Hamilton, Ontario Canada; 6 Department of Palliative Care, St Joseph’s Healthcare, Hamilton, Ontario Canada; 7 Department of Anesthesia, McMaster University, Hamilton, Ontario Canada

**Keywords:** Critical care, Quality improvement, Dying, Death, Palliative care, Spiritual care, Scale-up, Sustainability

## Abstract

**Background:**

Scaling-up and sustaining healthcare interventions can be challenging. Our objective was to describe how the 3 Wishes Project (3WP), a personalized end-of-life intervention, was scaled-up and sustained in an intensive care unit (ICU).

**Methods:**

In a longitudinal mixed-methods study from January 12,013 - December 31, 2018, dying patients and families were invited to participate if the probability of patient death was > 95% or after a decision to withdraw life support. A research team member or bedside clinician learned more about each of the patients and their family, then elicited and implemented at least 3 personalized wishes for patients and/or family members. We used a qualitative descriptive approach to analyze interviews and focus groups conducted with 25 clinicians who cared for the enrolled patients. We used descriptive statistics to summarize patient, wish, and clinician characteristics, and analyzed outcome data in quarters using Statistical Process Control charts. The primary outcome was enrollment of terminally ill patients and respective families; the secondary outcome was the number of wishes per patient; tertiary outcomes included wish features and stakeholder involvement.

**Results:**

Both qualitative and quantitative analyses suggested a three-phase approach to the scale-up of this intervention during which 369 dying patients were enrolled, having 2039 terminal wishes implemented. From a research project to clinical program to an approach to practice, we documented a three-fold increase in enrolment with a five-fold increase in total wishes implemented, without a change in cost. Beginning as a study, the protocol provided structure; starting gradually enabled frontline staff to experience and recognize the value of acts of compassion for patients, families, and clinicians. The transition to a clinical program was marked by handover from the research staff to bedside staff, whereby project catalysts mentored project champions to create staff partnerships, and family engagement became more intentional. The final transition involved empowering staff to integrate the program as an approach to care, expanding it within and beyond the organization.

**Conclusions:**

The 3WP is an end-of-life intervention which was implemented as a study, scaled-up into a clinical program, and sustained by becoming integrated into practice as an approach to care.

## Contributions to the literature

- Scaling-up and sustaining health interventions is challenging, even when worthwhile to patients, practitioners, or the public.
- For an end-of-life intervention with the aim of eliciting and implementing terminal wishes for dying critically ill patients, we documented the transition from research project to clinical program, to approach to practice. This was supported by a three-fold increase in enrolment with a five-fold increase in total wishes implemented, without a change in cost.
- Our analysis contributes to knowledge gaps surrounding the integration of interventions into clinical practice, providing insight into the importance of context, staff partnership and family engagement.

## Background

Understanding what matters most [1] to patients can guide goals of care discussions in the intensive care unit (ICU). Awareness of these values is paramount to ensure that end-of-life care is congruent with patient preferences. Conversations to learn more holistically about patients and their families are crucial to integrate the principles of palliative care into critical care.

Starting with such conversations, the 3 Wishes Project (3WP) involves eliciting and implementing terminal wishes to honor dying patients and comfort those attendant to the death [2]. Wishes are usually simple (e.g., a favorite beverage, spiritual or secular rituals, surrounding a patient with the people and things they love). The 3WP [3] began as a pilot project in a medical-surgical ICU in January 2013, and has been adopted and adapted in several North American centers [4].

Whether healthcare interventions are introduced through research or quality improvement initiatives, scaling up and sustaining them is challenging, even when worthwhile to patients, practitioners, or the public [5]. Program scale-up and sustainability are common terms in implementation science, with variable definitions [6, 7]. Aligned with the World Health Organization, our definition of scaling up is deliberate efforts to increase the local impact of a successfully developed intervention to benefit more individuals and foster program growth [8]. Aligned with recent reviews, sustainability is defined as continued delivery of a program while continuing to produce benefits following the duration of the research [7, 9].

The objective of this study was to describe how, over 6 years, the 3WP was scaled-up from a research project to become embedded as a widespread approach to end-of-life practice.

## Methods

### Ethics

We obtained ethics approval from the Hamilton Integrated Research Ethics Board (SJH REB 12–3776 [3 Wishes Pilot Project] and HIREB-1562 [3 Wishes Multicenter Evaluation]). Consent to participate in the clinical aspect of this project was verbal from patients and families, recorded by a bedside clinician in the patient’s chart. As the 3WP acknowledged naturally occurring aspects and extensions of current care, written consent was not necessary to offer this individualized bedside approach; however, written informed consent was obtained for all interviews and focus groups.

### Setting

The 3WP protocol was developed at St. Joseph’s Healthcare Hamilton in a 21-bed tertiary care ICU, initially implemented 1 week per month from January 2013–November 2014 [2]. Dying patients and families were invited to participate if the probability of dying was > 95% or after a decision to withdraw life support. A research team member or bedside clinician learned more about each of the patients and their family, then elicited at least 3 wishes from patients, family members and/or their clinicians, and implemented these wishes.

In the first report involving 40 patients and their families, we showed how this individualized approach to end-of-life care recognized the inherent dignity of dying patients, supported grieving families, and promoted humanism in practice [2]. Interest in this project grew, and the research team expanded the project’s scope into a clinical program offering it to all dying patients in the ICU. After 5 years, a multicenter formative evaluation demonstrated that the 3WP was transferable, affordable, sustainable and valuable for families, clinicians and organizations [10].

### Study design

In this report, we describe the history and current state of the 3WP in a mixed-methods embedded longitudinal study [11] in the original 3WP center.

### Data collection

We prospectively collected data on patients enrolled from January 1, 2013 - December 30, 2018. We recorded patient characteristics (e.g., age, admitting diagnosis, illness severity, wishes, and process-of-care variables, and date of death).

Quantitatively, we selected outcomes to evaluate the uptake, community engagement, and sustainability. Our primary outcome was the proportion of decedents enrolled per quarter; secondary outcome was the number of wishes; tertiary outcomes included whose wishes were elicited and who implemented the wishes (patient and/or family, clinicians or 3WP team); cost of wishes, and donated or discounted wishes.

Qualitative data were from individual interviews and focus groups with clinicians from January 2013–September 2018. We purposively sampled clinicians who cared for enrolled patients in their final 72 h. For interview and focus group participants, we recorded age, sex, relationship to patients (families), and duration of ICU experience (clinicians). We used a qualitative descriptive approach to analyze clinician perspectives and generate a rich description of the evolution of this intervention with minimal interpretation [12].

### Analysis

Quantitative data were reported using descriptive statistics such as absolute count (proportion), mean (standard deviation). We analyzed data in quarters using Statistical Process Control (SPC) Charts. An SPC chart is a graphical display of measurement over time which is useful in determining whether there is a change in process [13]. Starting from the inception of the project, a trend line based on the median aggregate quarterly enrollment served as reference for measuring project evolution. Serial data points were assessed in relation to the trend line and areas of ‘special cause variation’ were identified when data exhibited non-random patterns. These time periods were assessed in relationship to key implementation milestones to determine fundamental phases of the project [14]. We then analyzed data to report per phase the proportion of decedents who were enrolled; mean number of wishes implemented; proportion of wishes originating with patients and/or families or clinicians; proportion of wishes implemented by patients and/or families, clinicians or 3WP research team members; mean material cost of each wish; and proportion of wishes that were donated or discounted.

Qualitative content analysis was also used to understand scalability and sustainability. Initial transcripts were coded independently by one qualitative research associate (AT). Other investigators (MV, DJC, MS, NH, FJC) with qualitative research experience and clinical expertise (medicine, nursing, respiratory therapy) reviewed findings, progressively refining coding. Data collection and analysis occurred iteratively, with preliminary analysis from initial interviews shaping future data collection as this longitudinal project progressed.

### Role of the funding sources

The Hamilton Academy of Health Sciences, the Hamilton Chapter of the Canadian Intensive Care Foundation, the Greenwall Foundation and the Canadian Institutes for Health Research had no role in the design, conduct, or reporting of this study.

## Results

**Fig. 1:**
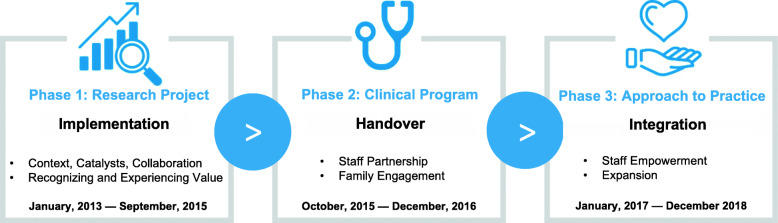
Three-phased approach to scale-up and sustainability of the 3 Wishes Project. Legend: The three phases of the 3 Wishes Project and key components of each phase are shown here

In both quantitative and qualitative data, we identified 3 phases (Fig. 1). First, the *research project* was scaled-up to become a *clinical program*, then became a general *approach to practice*. Key components in each phase facilitated these transitions. In the *research project* phase, a) context, catalysts and collaboration set the stage; b) experiencing and recognizing the project’s value facilitated transition to a clinical program. The *clinical program* involved a) staff partnership; and b) family engagement, which fostered program growth and transition to an approach to practice. The *approach to practice* phase was characterized by a) staff empowerment to independently initiate the program, and b) expansion beyond the dying process, the ICU and host organization.

### Quantitative results

#### Patients

From January 1, 2013 - December 31, 2018, 369 patients were enrolled, age 68.1 (15.5) years; most had medical admitting diagnoses (Table 1). Overall, 2039 wishes were implemented (Table 2).

**Table 1:** Characteristics of 369 Patients in the 3 Wishes Program

Baseline Characteristics	SJHH
Age, mean years (SD)	66.7 (15.6)
Female, n (%)	182 (49.3)
White race, n (%)	322 (87.3)
APACHE II score, mean (SD)	28.9 (8.8)
Admission type, n (%)	
Medical	339 (91.9)
Surgical	30 (8.1)
Spiritual belief, n (%)	
Christian (and variations)	208 (56.4)
Islam	4 (1.1)
Hindu	4 (1.1)
Buddhist	7 (1.9)
Sikh	3 (0.8)
Jewish	2 (0.5)
Agnostic	15 (4.1)
Spiritual	5 (1.4)
Other	2 (0.5)
None indicated	119 (32.2)
Reason for enrolment, n (%)	
Poor prognosis	217 (58.8)
Decision to withdraw advanced life support	152 (41.2)
**Characteristics During the ICU Stay**	
Advanced life supports administered any time in ICU, n (%)	
Mechanical ventilation	330 (89.4)
Inotropes	241 (65.3)
Dialysis	66 (17.9)
Advanced life supports withdrawn antemortem, n (%)	
Mechanical ventilation	214 (58.0)
Inotropes	71 (19.2)
Dialysis	11 (3.0)
Spiritual Care consult in ICU, n (%)	277 (75.1)
Palliative Care consult in ICU, n (%)	68 (18.4)
Organ Donation consult in ICU, n (%)	23 (6.2)

Legend: In this table, we describe the characteristics of 369 patients enrolled in the 3 Wishes Project at St. Joseph’s Healthcare Hamilton

**Table 2:** Number, Type and Cost of Wishes Implemented

**Wish Implementation**	
Average number of wishes/patient, n (SD)	5.5 (2.2)
**Categorization of Wishes**	
Facilitating Connections	216 (10.7)
Providing Food & Beverages	93 (4.6)
Personalizing the Environment	348 (17.2)
Humanizing the Patient	111 (5.5)
Music	179 (8.8)
Family Care	280 (13.8)
Rituals and Spiritual Support	263 (13.0)
Preparations and Final Arrangements	193 (9.5)
Word Clouds	170 (8.4)
Keepsakes & Tributes	87 (4.3)
Organ & Tissue Donation	24 (1.2)
Paying it Forward	53 (2.6)
Miscellaneous	8 (0.4)
**Cost of Wishes**	
Average cost/wish n, (SD)	8.06 (25.40)
Wishes that benefitted from a donation, n (%)	370 (18.7)
Wishes that cost 3WP nothing to implement, n (%)	1603 (80.9)

Legend: In this table, we describe 1982 terminal wishes implemented for 369 dying critically ill patients at St. Joseph’s Healthcare Hamilton

US exchange rate on May 24, 2019 = 1.34CAD

Cost of wishes based on completed wishes, total of 3407 wishes

#### Primary & secondary outcomes

**Fig. 2:**
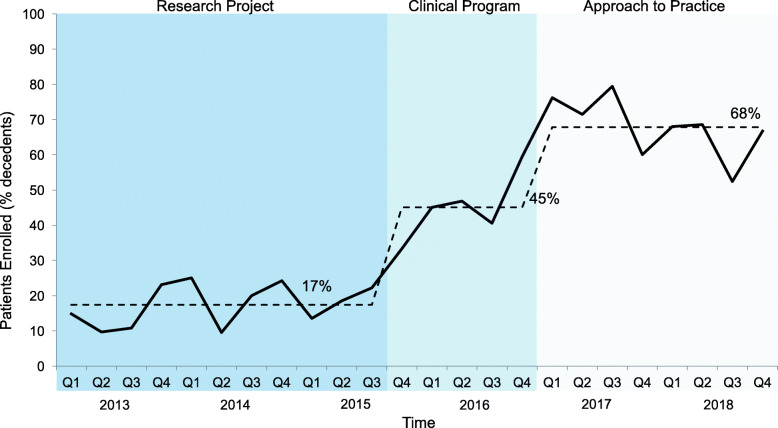
Proportion of dying patients enrolled per quarter. Legend: Percentage of patients enrolled in 3 Wishes Project of total ICU decedents per quarter. Mean percentage in each phase (research project, clinical program, and approach to practice) shown by dotted line

**Fig. 3:**
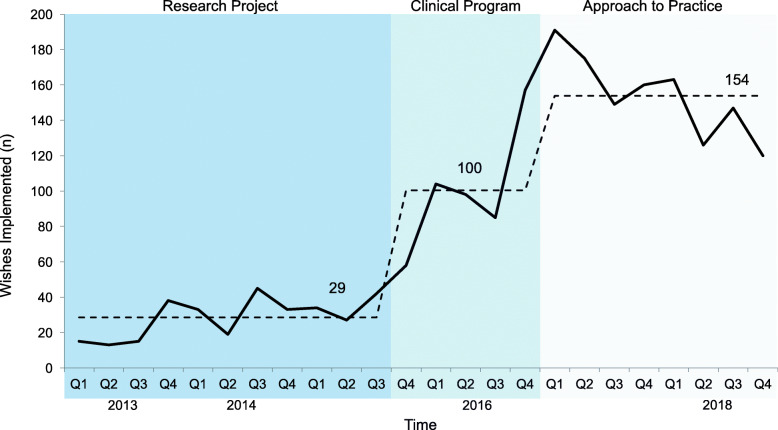
Total wishes per quarter. Legend: Number of wishes implemented per quarter. Mean number of wishes in each phase (research project, clinical program, and approach to practice) shown by dotted line

As a *research project* between January 1, 2013 - September 30, 2015, the project occurred during weeks when the principal investigator was the attending physician. The enrollment of patients was stable and no special cause variation was noted. In the *clinical program* phase, scope expanded by handover to the bedside staff who considered all dying patients eligible for enrollment, associated with special cause variation with 2 of 3 points noted above 2 sigma [14] of the research project phase. In the final phase, when the 3WP was integrated as an *approach to practice*, another period of special cause variation in patient enrollment was identified with 8 data points above the center line noted in the clinical program phase. (Fig. 2) A similar pattern was noted in the number of wishes implemented over time with corresponding periods of special cause variation (Fig. 3).

#### Tertiary outcomes

**Fig. 4:**
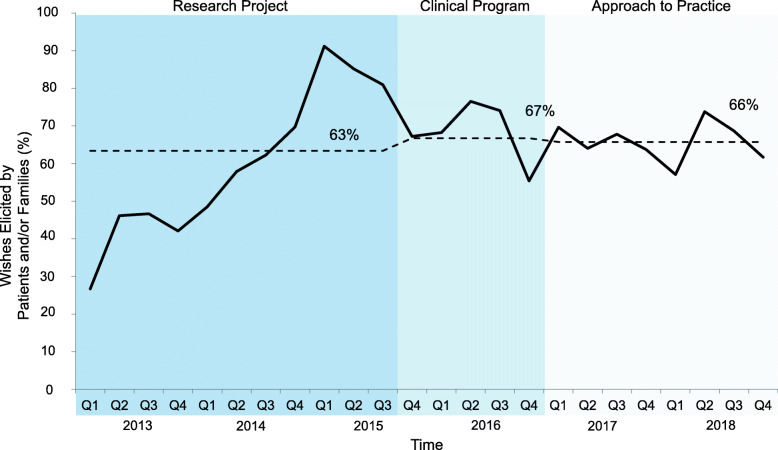
Proportion of wishes originating from patients and/or family members per quarter. Legend: Percentage of wishes elicited by the patient and/or family of total wishes elicited per quarter. Mean percentage in each phase (research project, clinical program, and approach to practice) shown by dotted line

**Fig. 5:**
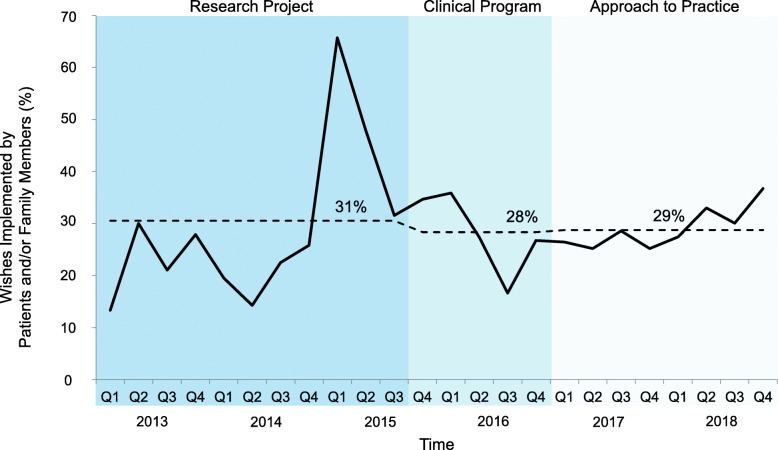
Proportion of wishes implemented by patients and/or family members per quarter. Legend: Percentage of wishes implemented by the patient and/or family of total wishes implemented per quarter. Mean percentage in each phase (research project, clinical program, and approach to practice) shown by dotted line

**Fig. 6:**
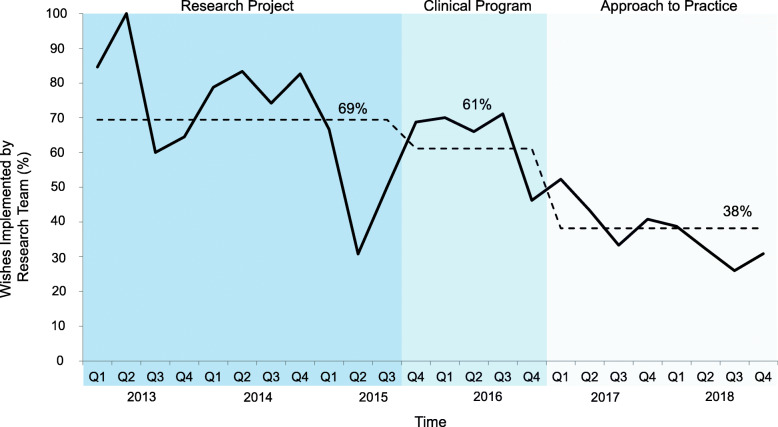
Proportion of wishes implemented by research team per quarter. Legend: Wishes implemented by 3 Wishes research team of total wishes implemented per quarter. Mean percentage in each phase (research project, clinical program, and approach to practice) shown by dotted line

**Fig. 7:**
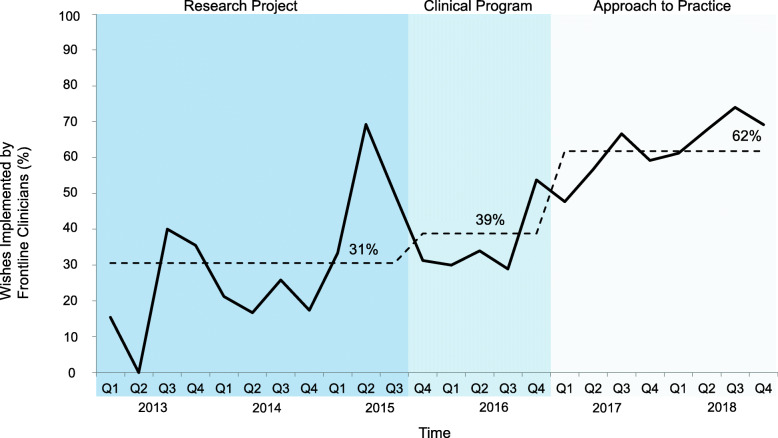
Proportion of wishes implemented by frontline clinicians per quarter. Legend: Percentage of wishes implemented by frontline clinical staff (i.e. RN, MD, Other ICU based clinicians) of total wishes implemented per quarter. Mean percentage in each phase (research project, clinical program, and approach to practice) shown by dotted line

The wishes were most often those of the patient and/or family – stable across all 3 phases – 63, 67 and 66%, respectively (Fig. 4). The implementation of wishes by patients and/or families was also similar across the phases (31, 28 and 29%) (Fig. 5). However, there was a shift in the other stakeholders who implemented wishes; most were enabled by the 3 Wishes team in both the research project (69%) and clinical program (61%) phases, decreasing to 38% in the approach to practice phase (Fig. 6). Wish implementation by frontline staff correspondingly increased over time in these 3 phases (from 31, 39 to 62%, respectively – primarily by bedside nurses) (Fig. 7).

**Fig. 8:**
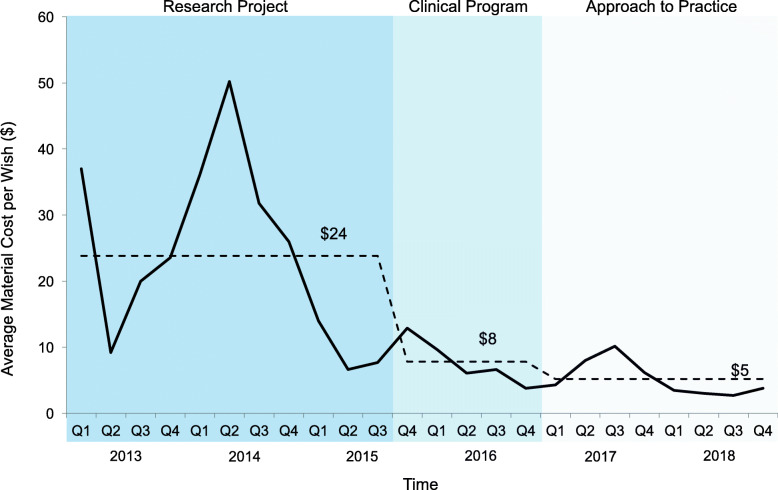
Mean material cost of each wish per quarter. Legend: The cost of materials required to implement each wish per quarter. Overall mean cost per wish for each phase (research project, clinical program, and approach to practice) shown by dotted line

**Fig. 9:**
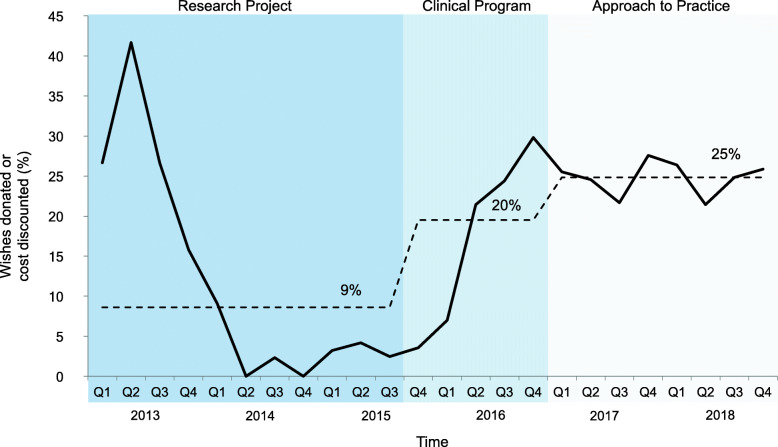
Proportion of donated or discounted wishes per quarter. Legend: Percentage of wishes supported by either donation (no cost) or discount (reduced cost) of total wishes. Mean percentage in each phase (research project, clinical program, and approach to practice) shown by dotted line

Finally, the mean cost/wish decreased over the 3 phases ($24/wish, $8wish and $6/wish, respectively) (Fig. 8), made partially possible through an increasing proportion of wishes supported by donations and/or discounted items (9, 20 and 25%, respectively) (Fig. 9), rendering material costs stable longitudinally despite a substantial increase in patient enrolment and wishes implemented.

### Qualitative results

We included 11 transcripts (5 focus groups and 6 interviews) eliciting the perspectives of 24 clinicians (2 spiritual care clinicians, 11 nurses, 5 residents, 1 respiratory therapist, 2 intensivists, 2 palliative care physicians), and 1 research staff.

### Phase 1: research project

Between January 2013–September 2015, the 3WP was implemented exclusively 1 week per month and patients were enrolled according to research protocol eligibility criteria. Slow, supported enrollment helped to maintain protocol fidelity while ensuring that authentic individualized end-of-life care could be provided.

- *Context, Catalysts, Collaboration*

As a non-technical intervention, the 3WP was led by a collaborative team of multidisciplinary clinicians from critical care, palliative care, and spiritual care. The investigators were identified as project catalysts, responsible for initiating the 3WP for eligible patients meeting research inclusion criteria. Drawing on their own clinical end-of-life care experience and skills, sometimes project catalysts shared strategies, introducing the project together in pairs,

*“It’s been nice to have [spiritual care clinician]. We’ve been together. And I remember [2 palliative care physicians] saying once [that] they had each other. Sometimes they would go in together in the difficult situations while I’d be knocking on [spiritual care clinician’s] door, you know? Because sometimes she has the right thing to say, and I have the right thing to say, and it just works.”*

[Research Coordinator].

Starting as a peer-review funded study engendered a scholarly orientation. The gradual introduction of the 3WP as a research project implemented 1 week/month focused on pursuing the project’s fundamental objective, described by a resident as, *“learning about the person and understanding what would be important to them”* [Resident]. The human connections formed in doing so helped to personalize the wishes. One nurse described her perspective,

*“I think it’s one of the studies that very quickly … people forgot it was a study because it had so much meaning. It had a different kind of impact on practice than something that’s a randomized controlled trial or a particular drug.”*

[Nurse].

b) *Experiencing and Recognizing Value*

As the research phase continued, frontline staff became more familiar with the project. Catalysts periodically shared stories about patients and families, creating a sense of community. Sharing the findings from staff focus groups provided opportunities to discuss the impact of the research project with peers. Clinicians began requesting the project for their dying patients as they perceived the positive influence of the 3WP, recognizing its value for families. One nurse described,

*“It is interesting how sometimes at the beginning … you feel kind of awkward bringing it up because it is a conversation about death and dying and palliation … but I think, once the family understands the goals of care, they’re so thankful for it [3WP] and then … you can see their comfort and their ease in their mind and in their eyes … and you feel like … you’re helping them a lot in their process.”*

[Nurse].

The research phase also involved post-mortem family interviews [2, 10]; sharing views of grief-struck relatives allowed staff to learn directly about the influence of the 3WP on families.

### Phase 2: clinical program

From October 2015–December 2016, clinical staff began to approach the research team for support implementing the project themselves for any dying patients. This handover from research staff to clinical staff was facilitated by staff partnership and family engagement.

- *Staff Partnership*

The research team’s role gradually transitioned from initiating the intervention to advising and supporting staff, through activities such as creating a Start-up Manual and resource binder, and updating staff on ongoing results in periodic newsletters and lunch-and-learn sessions. The unit communication clerks were invited to coordinate having staff sign sympathy cards. One catalyst described the project handover to frontline staff,

“*I would go to the bedside and say, ‘Oh are you comfortable? Would you like me to go with you?’ Or, ‘do you have that rapport?’ … so as it transitioned from research to program, I saw myself just backing away further and further, but gauging who I was interacting with … so giving others the opportunity to own it … but be there.”*

[Research Coordinator].

Early project catalysts began to instill a shared sense of ownership for the project as it transitioned to be more of a clinical program. Catalysts identified early adopters to role-model implementation through their clinical leadership. These champions were encouraged to take on increased responsibility, generating momentum among frontline staff. Champions led nursing huddles and reminded staff about resource cupboards and visual cues to increase awareness about dying patients, such as door magnets with the 3WP logo to signify dying patients. Champions shared experiences receiving one-to-one mentorship from catalysts. One nurse described,

“*… Just being in the room with someone, for the first time, that has had this discussion with families, you know, countless times before … just hearing the way the conversation was starting … it really allowed me, in further conversations with other families, to feel comfortable in bringing that point up.”*

[Nurse].

Another nurse echoed, *“I think there was a lot of value in me kind of learning the conversation and learning how to open up the conversation and that allowed me to help … progress with the way I would communicate with families in the future.”*

[Nurse].

b) *Family Engagement*

Enhanced engagement of family members further broadened the base of the 3WP. As clinical staff gained comfort introducing the project, they developed the confidence and communication skills to invite relatives to initiate or request more personalized wishes for their loved one, asking questions such as, “*who were they … what did they love to do?*” [Nurse]. They encouraged families to reminisce about the identity of their loved one, dwelling in the positive memories rather than exclusively focusing on the loss. This intentional approach created “*shared experiences*” [Spiritual Care Clinician] between patients’ families and clinicians. One nurse described how introducing a Word Cloud exercise engaged relatives,

*“The option of offering the Word Cloud is just so big. Like, it’s such a personal thing and at the bedside, it gives them a reason to write things down and reflect on funny stories and you in turn, learn things about that person. It just makes the whole thing more personal.”*

[Nurse].

Families also had practical suggestions such as creating a website [3]. Post-mortem family engagement included spontaneous phone calls, cards, emails, and texts to the staff and project team; unplanned family return visits (to debrief, request counselling, or reconnect with staff); and unsolicited donations from relatives to the 3WP. A resident described a special family connection created through the 3WP, when at the patient’s funeral, *“The mother - she requested that I sit next to her in the front row.”* [Resident].

### Phase 3: approach to care

From January 2017–December 2018, the 3WP was no longer considered an intervention with strict enrolment criteria or protocolized steps, but rather integrated as a common approach to care. Staff felt empowered to present the 3WP organically in spontaneous conversation with families, often without labelling the project or citing its name. Expansion occurred to other aspects of practice and elsewhere inside and outside the organization.

- *Staff Empowerment*

Through building capacity in frontline staff, staff became more empowered and skilled to provide individualized palliative care to dying patients and their families.

*“3 Wishes has, personally, made me feel empowered and confident to be my patients’ advocate and, first and foremost, for them, and for their family … [3 Wishes] gives me the courage to steer them in that direction to think about what would they really want.”*

[Nurse].

In contrast to previously needing instruction and confirmation from project catalysts to enroll patients, staff became increasingly comfortable eliciting and implementing wishes for dying patients as part of their practice, with physicians sharing terminal wishes on rounds, and incorporation into the clinical chart**.**

*“Now, I find it’s part of how we provide end-of-life care - mainly how the nurses provide end-of-life care. I … find those that didn’t initiate it in the beginning are now making note of it in their notes.”*

[Research Coordinator].

One nurse described the project as *“Bringing consistent value to death”.* [Nurse], describing how the 3WP has become an adaptable framework to personalize end-of-life conversations in the unit. Clinicians described using their clinical judgment and experience to independently care for patients this way*, “Because I work straight nights I don’t have people to go to, to ask, ‘Can we start this program?’ So, I usually bring it up myself.”* [Nurse]

b) *Expansion*

The spirit of the original research project now extends to a general approach to care by clinicians looking after patients who are not dying - stable and recovering patients as well. Activities that humanize the environment are more prevalent, regardless of a patient’s trajectory, such as creating personal playlists, or bringing flowers to a patient’s room pre-operatively, reflected in this sentiment: *“Well, why do we only have to do that when people are dying?”* [Nurse].

Staff now seek opportunities to implement the 3WP for patients throughout the hospital, prompting the creation of a new position – The 3 Wishes Expansion Coordinator, with the purpose of supporting the implementation 3WP throughout the hospital. The project’s longevity led to community awareness outside the ICU, such that families who previously experienced the 3WP and return to hospital began requesting the 3WP.

*“We’re at a point where … families who’ve had a loved one die in the ICU who were in 3 Wishes are now, again, in the hospital with someone else that’s dying, and they actually reach out to the staff and ask [if] 3 Wishes can be involved.”*

[Research Coordinator].

New norms have been established involving others in the organization who facilitate wishes. Decisions have sometimes created exceptions to the rules, such as off-season access to the hospital’s spiritual garden or temporarily lifting the institutional firewall of a social media site:

*“She was a young woman who wanted to update her Facebook page before she died and so there’s a firewall of course so people can’t go on Facebook and YouTube and things in the hospital, so they turned it off for the entire weekend - the IT people - [so she could] update her Facebook.”*

[Physician].

## Discussion

In this longitudinal study, the qualitative and quantitative analyses suggested a 3-phase approach to scaling up and sustaining this end-of-life care project. Beginning as a research study, the initial protocol provided structure. Authentic acts of compassion were ensured by a gradual approach to implementation, allowing frontline staff to experience and recognize the value of the intervention for patients, families and clinicians. The handover from research staff to bedside staff marked the transition to a clinical program, wherein staff partnership was characterized by one-on-one mentoring of project champions by project catalysts, and family engagement increased. The transition to an approach to care was characterized by staff empowerment to initiate and maintain the program, and expansion beyond end-of-life care, to the broader organization, and elsewhere.

Our quantitative results also reflect successful scale-up of this low-cost, high-impact personalized end-of-life program. A three-fold increase in enrolment from the research project phase to integration into clinical practice was associated with a five-fold increase in total wishes implemented, without a change in cost. This pattern was associated with special cause variation indicating process change, which paralleled our qualitative findings. Correspondingly, we observed a downward trend in research staff involvement over time, lending support to the 3WP as a sustainable program with minimal expenses, integrated into clinical workflow with increasing frontline staff leadership.

Scaling-up an intervention may require several strategies such as preparing the environment for change, assessing or increasing readiness for change, stakeholder collaboration, developing implementation strategies, and centralized monitoring and data analysis [15]. For this project, a common sense of purpose amongst interprofessional leaders sparked this structured yet individualized approach to end-of-life care, fueled by sharing narratives and research findings with staff, leading to enhanced recognition of the program’s value. The collaborative interdependent practice model [16] and non-hierarchical leadership enabled mentored staff champions to subsequently train others, who increasingly felt comfortable eliciting and implementing wishes. Growth was fostered through recognizing personhood and promotion of patient, family, and clinician agency.

Considering that few research-initiated health interventions are successfully sustained beyond the research phase [17], our analysis provides insight into factors which may support integration of an intervention into clinical practice. Rather than a series of discrete steps events, our findings reflect how scaling is context-dependent, and has formal and informal influences, shaped by peer involvement. It is noteworthy that the palliative care service was consulted for less than 20% of dying patients as staff became more empowered about this aspect of their practice. Our findings are consistent with the literature which cite program funding, stakeholder participation, and manageable workload as key factors associated with healthcare program sustainability [18]. Interviews confirmed how sustainability was fostered through staff partnership, creating shared ownership, followed by staff leadership which enhanced family participation eliciting and implementing wishes.

Patient and family engagement is emerging in critical care with untapped potential to enhance healthcare delivery [19]. In terms of setting research priorities, patients and relatives tend to focus on improving care provision, rather than acute interventions or innovations [20]. Facilitating authentic partnerships between relatives and staff is a fundamental way that the 3WP supports dying patients and their family. Heeding the call to develop deeper levels of engagement [21], we welcomed family-initiated post-mortem connections, encouraged family attendance at quarterly hospital memorial services, and held a Celebration of Life event. In turn, families encouraged us to continue the 3WP through periodic visits and correspondence, donations (e.g., knitting, participating in fundraising events), and speaking at a health quality conference.

Strengths of this study include clinician perspectives gathered over time which describe the program’s evolution and longevity, as well as standard approaches including SPC methods to quantitatively analyze stability, change, and assessment of trends. Increasing participation rates demonstrated the project expansion from a research-based initiative to a unit-based approach to practice. Limitations include the retrospective analysis and single-center experience. As family interviews on the 3WP are cross-sectional rather than longitudinal [2, 10], family views are not directly represented again in this report. Interventions such as this may not be sustained in settings where practice improvements are not valued in the domain of palliative care, or where research and clinical staff do not collaborate as effectively.

Early consistent family feedback that the 3WP helped them emotionally to weather the transition from curative to comfort-oriented care was motivational. As expressed by a patient’s nephew,

*“Full steam ahead. Don’t give up. Never give it up. If you can do it... don’t ever give it up... because if you give it up then you’re just going to be just a standard hospital all over again where people die, and nothing extraordinary will come from here. And when something extraordinary is in the making you don’t give it up. If you were to take this program away, you would be doing a disservice to the people who are dying in there … because I think it helps. You know? Simple things. Simple gestures. And that’s all you need.”*

[Family Member].

## Conclusions

The care of dying critically ill patients in the 3 Wishes Program has demonstrated scalability and sustainability over 6 years, transitioning over 3 phases from a research project to a clinical program and to approach to practice.

## Data Availability

The qualitative datasets generated during the current study are not publicly available due to the potentially identifying nature of the complete qualitative transcripts and lack of consent from participants to publicly share this data. Quantitative datasets used and analysed during the current study are available from the corresponding author on reasonable request.

## Abbreviations

ICU: Intensive Care Unit
3WP: 3 Wishes Project
SPC: Statistical Process Control
